# Acknowledgement

**Published:** 2022

We are extremely grateful to the following members of the medical profession from Pakistan and Overseas for sparing their precious time to review the manuscript during the year, 2021.

Aatik Arsh, **Peshawar** 7

Abdul Hannan, **USA** 1

Abdul Haque, **Faisalabad** 1

Abdul Qadeer Memon, **Saudi Arabia** 2

Abdul Razaque Shaikh, **Karachi** 4

Abdul Samad Khan, **Saudi Arabia** 1

Abdullah AlAlwani, **Turkey** 1

Abdullah Boyuk, **Turkey** 1

Abid Saleem, **Karachi** 1

Abida Shaheen, **Islamabad** 1

Abrar Ahmed Javed, **Multan** 2

Abrar A. Wagan, **TM Khan** 2

Afifa Ehsan, **Lahore** 1

Afshan Shahid, **Lahore** 1

Afzal Javed, **UK** 1

Ahmed Bader, **Saudi Arabia** 1

Ahmet Balik, **Turkey** 2

Ahmet Sarici, **Turkey** 1

Ahsan Mukhtar, **UK** 1

Ahsan Sethi, **Peshawar** 2

Ajwad Farogh, **Bahawalpur** 1

Ali Dogan, **Turkey** 1

Ali Eser, **Turkey** 1

Ali Madeeh Hashmi, **Lahore** 2

Alia Halim, **Islamabad** 1

Aliya Hisam, **Rawalpindi** 2

Ambreen Usmani, **Karachi** 4

Ambrin Fatima, **Karachi** 1

Amina Ahmad, **Lahore** 2

Amjad Hussain Wyne, **Lahore** 1

Ammar Anwer, **Lahore** 1

Ammara Mushtaq, **USA** 3

Anisuddin Bhatti, **Karachi** 2

Anjum Jalal, **Rawalpindi** 1

An-Jun Li, **China** 1

Anmol Shahid, **Canada** 2

Ansa Rabia, **Lahore** 1

Arif Emre, **Turkey** 1

Arshad Hasan, **Karachi** 1

Arshad Hussain, **Peshawar** 1

Ashraf Ganatra, **Karachi** 1

Asim Shah, **USA** 1

Asmat Shaheen, **Kohat** 1

Asude Aksoy, **Turkey** 1

Ata Mahmoodpoor, **Iran** 3

Atia ur Rehman, **Lahore** 1

Attia Bari, **Lahore** 2

Awad M. Ahmed, **Sudan** 1

Ayman F. Alshayji, **Kuwait** 1

Azhar Rashid, **Karachi** 2

Aziz Fatima, **Lahore** 1

Bader F. Zuberi, **Karachi** 5

Bisma Ejaz, **Lahore** 1

Bobby Joseph, **India** 1

Burak Acikel, **Turkey** 1

Bushra Sher Zaman, **Bahawalpur** 1

Chen-li Zhang, **China** 1

Chong-Chao Yan, **China** 1

Chun-guo Wang, **China** 3

Chunying Zhu, **China** 1

Cihan Heybeli, **Turkey** 1

Col. Ch. Aqeel Safdar, **Islamabad** 1

Dilshad Ahmed Khan, **Rawalpindi** 1

Donald Hathaway III, **USA** 1

Ejaz Ahmed Vohra, **Karachi** 4

Elfadil Abass, **Saudi Arabia** 1

Enes Uyar, **Turkey** 1

Ertan Mert, **Turkey** 1

Esengul Unguder, **Turkey** 1

Esra Atilgan, **Turkey** 1

Fahd Haroon, **Karachi** 1

Fahim Vohra, **Saudi Arabia** 1

Faisal Nazeer Hussain, **Lahore** 6

Faiza Hassan, **Islamabad** 1

Faraz Shafiq, **Karachi** 4

Farhat Nadeem, **Lahore** 1

Farzana Ashraf, **Lahore** 1

Fatima Aamir Khan, **Wah Cantt** 2

Fatima Ali, **Lahore** 1

Fatima Naumeri, **Lahore** 1

Fazal I Wahid, **Peshawar** 1

Fazal-ur-Rehman, **Karachi** 1

Fizza S. Gillani, **USA** 1

Furhan Iqbal, **Multan** 1

Ghulam Asghar Channa, **Karachi** 2

Ghulam Sarwar Pirkani, **Quetta** 3

Gohar Wajid, **Egypt** 6

Guojin Zuo, **China** 1

Gurkan Ozturk, **Turkey** 2

H.R. Ahmad, **Karachi** 3

Haci Yusuf Gunes, **Turkey** 1

Hadred Matheos, **London** 1

Hai-bo Li, **China** 2

Haider Ghazanfar, **USA** 1

Haitham Osman, **Saudi Arabia** 1

Handan Inonu Koseoglu, **Turkey** 1

Haroon Tayyab, **Karachi** 1

Hasan Ejaz, **Saudi Arabia** 5

Henrico Badaoui Strazzi Sahyon, **Brazil** 2

Hongmei Zhao, **China** 3

Hongwei Zhang, **China** 2

Hong-xun Cui, **China** 2

Hong-ya Li, **China** 2

Hong-zhen Zhang, **China** 1

Huan-rong Feng, **China** 2

Huaxiang Xia, **China** 1

Huiling Xue, **China** 2

Huiyi Ye, **China** 3

Huma Mamun Mahmud, **UAE** 4

Huma Mushtaq, **Rawalpindi** 1

Humaira Zafar, **Islamabad** 10

Ibrahim Bashan, **Turkey** 1

Iftikhar Ahmad Jan, **UAE** 1

Ilhami Berber, **Turkey** 1

Imran Ijaz Haider, **Lahore** 1

Imtiaz Khalid, **Karachi** 4

Iqbal A. Muhammad Khyani, **Karachi** 2

Irfan Ullah, **Multan** 1

Jahanara Ainuddin, **Karachi** 2

Jamal Hafeez, **Multan** 1

Jiang Zheng, **China** 2

Jian-zhou Tong, **China** 1

Jiaywei Tsauo, **China** 1

Jinghui Dong, **China** 1

Jingqin Liu, **China** 3

Jinsheng Wang, **China** 1

John Outland, **USA** 1

Joseph Bosco, **USA** 1

Jun-kai Xu, **China** 1

Kaleem Ullah Toori, **Islamabad** 1

Kamran Hafeez, **Saudi Arabia** 3

Kamran Hafeez, **UK** 1

Kamran Sattar, **Saudi Arabia** 1

Khalid Ansari, **UK** 1

Khalid Mahmood, **Karachi** 2

Khalid Saeed Khan, **Spain** 1

Khemchand N Moorani, **Karachi** 1

Kostatinos Sergis, **Greece** 1

Kyung-Rae Kim, **USA** 1

Laima Alam, **Rawalpindi** 5

Lei Li, **China** 1

Liang Hong, **China** 1

Liaqat Ali Rao, **Multan** 2

Lin Li, **China** 1

Lina Peng, **China** 1

Li-na Shang, **China** 1

Ling Zhang, **China** 1

Ling-ling Li, **China** 1

Li-ping Du, **China** 2

Liu Jingqin, **China** 1

Lubna Meraj, **Rawalpindi** 2

M Akbar Chaudhry, **Lahore** 4

M Ibrahim Hasan, **Malaysia** 1

M Kamran, **Karachi** 2

M Naeem Afzal, **Lahore** 1

M Perwaiz Iqbal, **Lahore** 2

M Yahya Noori, **Karachi** 1

M Yasir Khan, **Multan** 4

M. Amir, **Karachi** 1

M. Asim Mehboob, **Gujranwala** 2

M. Aslam, **Lahore** 1

M. Azeem Aslam, **Islamabad** 1

M. Faheem Afzal, **Lahore** 1

M. Imran, **Multan** 1

M. Kamran, **Karachi** 3

M. Mujahid Khan, **Saudi Arabia** 1

M. Naeem Afzal, **Lahore** 1

Maj. Gen. M. Aslam, **R'pindi** 5

Mansoor Khan, **Islamabad** 1

Maryam Gul Agha, **Lahore** 1

Masood Ahmad Sheikh, **Karachi** 1

Masood Jawaid, **Karachi** 1

Mazhar Malik, **Islamabad** 1

Mazhar ul Hasan, **Karachi** 1

Md. Abdus Salam, **Malaysia** 2

Meharunnissa Khaskheli, **Hyderabad** 5

Mehmet Adam, **Turkey** 1

Mehmet Ferdi Kinci, **Turkey** 1

Mingyue Liu, **China** 1

Miray Cetinkaya, **Turkey** 1

Misbah Durrani, **Islamabad** 1

Mohamed Awad Mohamed Ismail, **Egypt** 1

Mohsin Ali, **USA** 1

Mostafa Kamel Al Fouly, **Egypt** 1

Mozaffar Rahim Hingorjo, **Karachi** 2

M. Abubakar, **Lahore** 1

M. Farooq, **Bahawalpur** 1

Muhammad, **Islamabad** 1

M. Shahid, **Multan** 1

Mujeeb Ur Rehman, **Karachi** 1

Mujeeb Ur Rehman, **Peshawar** 4

Mukhtiar Baig, **Saudi Arabia** 2

Mulazim Hussain Bukhari, **Muzaffarabad** 1

Munawar Alam Ansari, **Hyderabad** 4

Musarrat Riaz, **Karachi** 9

Mustafa Naseem, **Karachi** 2

Na Cui, **China** 4

Nadeem Ahmad, **Karachi** 3

Nadeem Iqbal, **Islamabad** 1

Narin Yildirim, **Turkey** 1

Naseer Ahmed, **Karachi** 1

Naseer Ahmed, **Peshawar** 1

Nasim Karim, **Karachi** 1

Nasir Khokhar, **Islamabad** 3

Nauman Tarif, **Lahore** 1

Nausheen Hayat, **Karachi** 2

Naveed Ashraf, **Lahore** 1

Nawal Al Mashaikhi, **Oman** 1

Nazish Imran, **Lahore** 6

Nazli Hossain, **Karachi** 2

Noor-i-Kiran Haris, **Faisalabad** 4

Nureddin Yuzkat, **Turkey** 1

Paul Sharpe, **UK** 2

Ping Wang, **China** 1

Qian Sun, **China** 1

Quanjin Si, **China** 2

Rabia Arshad, **Karachi** 1

Ramesh Kumar, **Islamabad** 6

Raner Lashom, **Denmark** 1

Rashida Parveen, **Multan** 6

Rehan A. Khan, **Islamabad** 3

Rehman Siddiqui, **Karachi** 1

Riffat Jaleel, **Karachi** 2

Rizwan Jouhar, **Karachi** 1

Romeeza Tahir, **Lahore** 1

Rongrong Ren, **China** 1

Roquyya Gul, **Lahore** 1

Rubina Naqvi, **Karachi** 4

Ruhsan Muduroglu, **Turkey** 1

Saba Sohail, **Karachi** 5

Sabri Selcuk Atamanalp, **Turkey** 1

Sadaf Iftikhar, **Lahore** 1

Sadananda Acharya, **Saudi Arabia** 1

Sadia Fatima, **Karachi** 1

Saera Suhail Kidwai, **Karachi** 1

S. Nasir Mansoor, **Okara** 4

Sahrai Saeed, **Norway** 4

Saima Askari, **Karachi** 3

Sajid Malik, **Islamabad** 1

Sajid Rashid, **Multan** 4

Salman A. Tipu, **Islamabad** 1

Samara Siddique, **Lahore** 1

Sameen Afzal Junejo, **Hyderabad** 3

Sami Saeed, **Rawalpindi** 1

Sana Jalil, **Rawalpindi** 1

Seema Bibi Qureshi, **Saudi Arabia** 3

Senol Demircan, **Turkey** 1

Servet Karagul, **Turkey** 1

Sevgi Ozcan, **Turkey** 1

SH Waqar, **Islamabad** 3

Shabeen Naz Masood, **Karachi** 1

Shabih H Zaidi, **UK** 2

Shah Jahan, **Lahore** 1

Shahid Hameed, **Lahore** 1

Shahid Sarwar, **Lahore** 6

Shahzeb Ahmed Satti, **Quetta** 1

Shankar Lal, **Ireland** 3

Shanshan Cai, **China** 2

Shanshan Zhu, **China** 1

Shao-hui Zhang, **China** 1

Shaukat Ali Jawaid, **Karachi** 7

Shazia Qayyum, **Islamabad** 1

Shehnoor Azhar, **Lahore** 2

Shoaib Saleem, **Multan** 1

Shouguo Fang, **China** 1

Shuli Li, **China** 1

Sohail A. Khan, **Karachi** 2

Stephen Onwere, **Nigeria** 2

Sultan A. Alshoabi, **Saudi Arabia** 6

Sultan Ayoub Meo, **Saudi Arabia** 2

S.Kaleem Ur Rahman, **Peshawar** 1

S. Mamun Mahmud, **UAE** 3

S. Najam Hyder, **Multan** 2

S. Nazim H. Bokhari, **Lahore** 3

S. Rashid Habib, **Saudi Arabia** 1

S. Riazul Hasan, **Karachi** 2

S. Rizwan ul Ahsan Jilani, **Lahore** 1

S. Wasim Akhtar, **Karachi** 1

S. Zulfiquar Ali Shah, **Hyderabad** 7

S. Hanaa Fatima, **Rawalpindi** 1

S. Kauser Ali, Karachi 1

Tahira Nishtar, **Peshawar** 1

Tahira Perveen Umer, **Karachi** 1

Tao Gu, **China** 1

Tareef S. Daqqaq, **Saudi Arabia** 1

Tariq Mahmood, **Karachi** 3

Tariq Wahab Khanzada, Oman 1

Tariq Waqar, **Lahore** 1

Tayyaba Gul Malik, **Lahore** 4

Tayyiba Wasim, **Lahore** 2

Ting-ting Li, **China** 1

Ubaid Ullah, **Multan** 1

Usman Anwer Bhatti, **Islamabad** 1

Usman Mahboob, **Peshawar** 4

Uzma Abdullah, **Rawalpindi** 1

Uzma Z. Khan, **USA** 1

Veoler Martine, **USA** 1

Waheed Khan, **Australia** 1

Wajid Jawaid, **Karachi** 12

Waqas Sami, **Lahore** 1

Waqas Tahir, **Canada** 2

Waris Qidwai, **Karachi** 5

Wasim Sattar, **Multan** 1

Wei Geng, **China** 3

Wei Wang, **China** 1

Xiang-ju Zhuge, **China** 1

Xian-yi Liu, **China** 1

Xiao Zhang, **China** 1

Xiao-fang Li, **China** 1

Xiaoguang Yu, **China** 2

Xin Li, **China** 1

Xin-xin Zhang, **China** 2

Xinyu Yang, **China** 1

Xiuhua Dai, **China** 2

Yanfeng Guo, **China** 1

Yang Liu, **China** 1

Yaqoob Ahmedani, **Karachi** 1

Yihui Wang, **China** 1

Ying Hu, **China** 4

Younis Abdelwahab Skaik, **Germany** 6

Yuanda Zhang, **China** 2

Yuanfu Wei, **China** 1

Yu-kun Luo, **China** 2

Yundai Chen, **China** 1

Yusuf Taha Gullu, **Turkey** 1

Yu-Xi Xie, **China** 1

Zafar Iqbal, **Peshawar** 1

Zahid Kamal, **Lahore** 1

Zheng Zhang, **China** 2

Zhen-yu Cui, **China** 1

Zhihong Li, **China** 1

Zhiqiang Ren, **China** 1

Zhi-Yong Bu, **China** 2

Ziauddin Shaikh, **UK** 1

Zilli Huma, **Peshawar** 1

Zohaib Akram, **Australia** 1

Zoya Zahid Ch, **Sialkot** 1

